# Mutation of self-binding sites in the promoter of the MrpC transcriptional regulator leads to asynchronous *Myxococcus xanthus* development

**DOI:** 10.3389/fmicb.2023.1293966

**Published:** 2023-11-23

**Authors:** Maeve McLaughlin, Penelope I. Higgs

**Affiliations:** Department of Biological Sciences, Wayne State University, Detroit, MI, United States

**Keywords:** *Myxococcus xanthus*, biofilm, genetic regulatory network, development, negative autoregulation

## Abstract

**Introduction:**

MrpC, a member of the CRP/Fnr transcription factor superfamily, is necessary to induce and control the multicellular developmental program of the bacterium, *Myxococcus xanthus*. During development, certain cells in the population first swarm into haystack-shaped aggregates and then differentiate into environmentally resistant spores to form mature fruiting bodies (a specialized biofilm). *mrpC* transcriptional regulation is controlled by negative autoregulation (NAR).

**Methods:**

Wild type and mutant *mrpC* promoter regions were fused to a fluorescent reporter to examine effects on *mrpC* expression in the population and in single cells *in situ*. Phenotypic consequences of the mutant *mrpC* promoter were assayed by deep convolution neural network analysis of developmental movies, sporulation efficiency assays, and anti-MrpC immunoblot. In situ analysis of single cell MrpC levels in distinct populations were assayed with an MrpC-mNeonGreen reporter.

**Results:**

Disruption of MrpC binding sites within the *mrpC* promoter region led to increased and broadened distribution of *mrpC* expression levels between individual cells in the population. Expression of *mrpC* from the mutant promoter led to a striking phenotype in which cells lose synchronized transition from aggregation to sporulation. Instead, some cells abruptly exit aggregation centers and remain locked in a cohesive swarming state we termed developmental swarms, while the remaining cells transition to spores inside residual fruiting bodies. *In situ* examination of a fluorescent reporter for MrpC levels in developmental subpopulations demonstrated cells locked in the developmental swarms contained MrpC levels that do not reach the levels observed in fruiting bodies.

**Discussion:**

Increased cell-to-cell variation in *mrpC* expression upon disruption of MrpC binding sites within its promoter is consistent with NAR motifs functioning to reducing noise. Noise reduction may be key to synchronized transition of cells in the aggregation state to the sporulation state. We hypothesize a novel subpopulation of cells trapped as developmental swarms arise from intermediate levels of MrpC that are sufficient to promote aggregation but insufficient to trigger sporulation. Failure to transition to higher levels of MrpC necessary to induce sporulation may indicate cells in developmental swarms lack an additional positive feedback signal required to boost MrpC levels.

## 1 Introduction

*Myxococcus xanthus* is a “social” bacterium with a life cycle that is highly dependent on collective behaviors (Munoz-Dorado et al., 2016). During vegetative growth, large groups of *M. xanthus* cells swarm over solid surfaces in search of prey on which they cooperatively predate (Rosenberg et al., 1977; Berleman et al., 2008). Under nutrient limited conditions, *M. xanthus* enters an ~72-h developmental program during which cells form a specialized biofilm and segregate into distinct cell fates (Higgs et al., 2014). Some cells are induced to swarm into aggregation centers to produce mounds of approximately 10^5^ cells. Once inside aggregation centers, individual cells slow down and stop moving (Cotter et al., 2017), which prevents them from leaving the aggregation center. These cells are then induced to differentiate into environmentally resistant spores, producing mature multicellular fruiting bodies. Other cells within the developing population remain outside of the fruiting bodies and differentiate into peripheral rods, which are thought of as a persister-like state (O’Connor and Zusman, 1991; Lee et al., 2012). For development to be effective, cells in the population must coordinate their behavior both in time and space. If sporulation were to occur before completion of aggregation, then the benefits conferred by a multicellular fruiting body structure, such as enhanced resistance to environmental stresses or group dispersal, would be lost (Velicer et al., 2014).

MrpC, is a CRP/Fnr superfamily transcriptional regulator that coordinates expression of hundreds of developmental genes (Sun and Shi, 2001b; Robinson et al., 2014). Under standard laboratory developmental conditions, no ligand has been identified to activate MrpC, and purified MrpC binds efficiently to target DNA binding sites *in vitro* (Nariya and Inouye, 2006; Mittal and Kroos, 2009a; McLaughlin et al., 2018). Threshold levels of MrpC appear to drive distinct stages of development: low levels are associated with induction of aggregation onset, higher levels are associated with commitment to sporulation (Lee et al., 2012; Rajagopalan and Kroos, 2014, 2017; Hoang and Kroos, 2018). Peripheral rods contain low levels of MrpC in the wild type *M. xanthus* strain DZ2 (Lee et al., 2012). One immediate target of MrpC is activation of the transcription factor, FruA (Ueki and Inouye, 2003). FruA is essential for development (Ogawa et al., 1996; Ellehauge et al., 1998), and FruA and MrpC coordinately induce expression of multiple downstream genes (Mittal and Kroos, 2009b; Lee et al., 2011; Son et al., 2011).

MrpC is under complex regulation. Shortly after cells sense nutrient limitation, *mrpC* expression is upregulated by MrpB, a bacterial enhancer binding protein (bEBP) (Sun and Shi, 2001a). MrpB binds to two upstream activating sequences (UAS1 and UAS2) 182 bp from the *mrpC* start codon, where it presumably stimulates *mrpC* expression from a sigma^54^-dependent promoter (Sun and Shi, 2001a; McLaughlin et al., 2018). MrpC directly binds to at least four sites (BS1, 3, 4, and 5) in its own promoter and functions as a negative autoregulator by competing with its transcriptional activator, MrpB, for overlapping UAS1/BS3 and UAS2/BS4 binding sites (McLaughlin et al., 2018). MrpC also positively regulates expression of *mrpB* (Sun and Shi, 2001a) (C. Mataczynski and P.I. Higgs, unpublished results). Finally, early during the developmental program, gradual accumulation of MrpC is achieved because the Esp signaling system induces turnover of MrpC (Higgs et al., 2008; Schramm et al., 2012). Several additional post-transcriptional events modulate MrpC accumulation (and therefore progression through development) in response to changing environmental conditions (Nariya and Inouye, 2005; Rajagopalan and Kroos, 2014, 2017; Marcos-Torres et al., 2020).

NAR is a particularly abundant genetic regulatory network motif (Thieffry et al., 1998; Rosenfeld et al., 2002). Theoretical and experimental data have demonstrated NAR network motifs serve to buffer against transcriptional noise, speed up response times, increase the input dynamic range of a circuit, and optimize fitness (Becskei and Serrano, 2000; Rosenfeld et al., 2002; Camas et al., 2006; Nevozhay et al., 2009; Kozuch et al., 2020). Most of these well-described functions have been investigated synthetic systems or in single-celled organisms; fewer examples of the phenotypic consequences of perturbing network motifs in natural multicellular systems are available.

Here, we set out to investigate the role of MrpC NAR in the context of the multicellular developmental program in *M. xanthus*. We demonstrate that disruption of BS1 and BS5 independently and additively increase *mrpC* reporter expression, consistent with both playing a role in NAR. *mrpC* expressed from a BS1/BS5 mutant promoter was associated with early and increased MrpC accumulation and led to premature aggregation onset, reduced fruiting body organization, and, unexpectedly, reduced sporulation efficiency. Using a method to film strains developing under submerged culture (Glaser and Higgs, 2019), we observed that this strain exhibited striking asynchronous development: after formation of aggregates, some cells suddenly exited aggregation centers as fast-moving swarms, while other cells remained in stationary fruiting bodies. Deep convolution neural network analyzes indicated these developmental swarms displayed trajectories and velocities that were distinct from cells in the mobile aggregate phase. Also consistent with loss of NAR, analysis of single cell *mrpC* expression *in situ* demonstrated that mutated BS1/BS5 resulted in increased cell-to-cell variability of *mrpC* expression. These data suggest that in the context of the multicellular developmental program, MrpC NAR may help to constrain variation in *mrpC* expression within the developing population to facilitate synchronized transition from cells in the aggregation state to the sporulation state. Interestingly, *in situ* analysis of single cell MrpC-reporter accumulation suggested that developmental swarms maintained MrpC levels intermediate between those found in peripheral rods and sporulating cells suggesting distinct MrpC steady-state levels can produce novel group phenotypes.

## 2 Materials and methods

### 2.1 Bacterial strains, plasmids, and oligonucleotides

The bacterial strains used in this study are listed in Supplementary Table S1. Plasmids were constructed (Lee et al., 2010) using the oligonucleotide sequences, and construction strategy listed in Supplementary Table S2.

### 2.2 Growth and developmental conditions

*Escherichia coli* were grown under standard laboratory conditions in LB media supplemented with 50 μg ml^−1^ of kanamycin and/or 20 μg ml^−1^ of tetracycline, where necessary (Maniatis et al., 1982). *M. xanthus* DZ2 strains were grown under vegetative conditions on CYE agar or in broth, as described previously (Lee et al., 2010); plates were supplemented with 100 μg ml^−1^ of kanamycin and/or oxytetracycline at 10 μg ml^−1^, where necessary.

*M. xanthus* strains were induced to develop under submerged culture conditions (Lee et al., 2010). Briefly, vegetative cells were diluted to an absorbance measured at 500 nm (A_550_) of 0.035 in fresh CYE broth, seeded into petri dishes or tissue culture dishes (as indicated in the relevant methods sections) and allowed to grow to a confluent layer for 24 h at 32°C. We estimate the cells grew to approximately 6 × 10^5^ cells mm^−1^ (Lee et al., 2010). To initiate development, CYE media was removed and replaced with an equivalent volume of MMC buffer [10 mM 4-morpholinepropanesulfonic acid (MOPS) pH 7.6, 4 mM MgSO_4_, 2 mM CaCl_2_], followed by continued incubation at 32°C for 72–120 h.

To record static developmental phenotypes, 0.5 mL diluted cells were seeded into 24-well tissue culture plates in triplicate and imaged at the indicated times with a Leica M80 stereomicroscope.

### 2.3 Analysis of mCherry fluorescence by plate reader

Submerged culture assays were set up using 0.5 mL diluted cells seeded into each well of 24-well tissue culture plates, and population mCherry fluorescence was measured as described previously (McLaughlin et al., 2018). Briefly, developing cells were harvested at the indicated hours post-starvation, dispersed, and 1/10 volume of each sample was assayed for fluorescence at 580 nm in a Typhoon imager scan. Values plotted are the average and associated standard deviation from three independent biological replicates. Similar patterns were observed when fluorescence was first normalized to total protein concentration from each sample at each time point (data not shown).

### 2.4 Sporulation assay

Developmental sporulation efficiency was determined as described previously from 0.5 mL diluted cells developed in triplicate in 24-well tissue culture plates under submerged culture conditions (Lee et al., 2010). Briefly, heat (50°C for 60 min)- and sonication (60 pulses 30% output)-resistant spores were enumerated in a Helber counting chamber. Sporulation efficiency was calculated as percent of wild type spores at 72 or 120 h as indicated. Values reported are the average and associated standard deviation from triplicate independent biological experiments. Chemical-induced sporulation was triggered by addition of glycerol to 0.5 M to vegetatively growing cells in CYE broth with shaking incubation for 24 h at 32°C (Muller et al., 2010). Spores were isolated and enumerated as indicated above.

### 2.5 Developmental video analysis

*M. xanthus* strains were induced to develop under submerged culture using 0.15 mL diluted cells per well in 96-well tissue culture plates. After induction of starvation, plates were incubated in a Tecan Spark10M plate reader preheated to 32°C (Glaser and Higgs, 2019). The center of each well was imaged every 30 min from 0 to 72 h post-starvation and images assembled into movies (6 fps) in ImageJ (Schindelin et al., 2012). For each movie, onset of aggregation (Agg_ONSET_), maximum aggregates (Agg_MAX_) and final fruiting bodies (Agg_FINAL_) were enumerated as described previously (Glaser and Higgs, 2019). The percent of aggregates that transitioned to stationary fruiting bodies was calculated as [Agg_FINAL_/Agg_MAX_]. The number of aggregates that were mobile (Agg_MOBILE_) in each movie was recorded and percent Agg_MOBILE_ was calculated as [Agg_MOBILE_/Agg_FINAL_]. For each movie, the first frame (Mobility_I_) and final frame (Mobility_F_) in which aggregates were mobile was recorded and mobility duration was calculated as [(Mobility_F_ – Mobility_I_) × 0.5 h/frame]. Mobility delay was calculated as [Mobility_I_ – Agg_ONSET_]. Data were compiled from three biological replicates that contained five technical replicates per strain. Statistical significance was analyzed in Prism (GraphPad) using unpaired *t*-test assuming Gaussian distribution, or otherwise the Mann–Whitney test.

### 2.6 Neural network training and analysis

For tracking aggregate and swarm mobility, DeepLabCut deep convolution neural network was used (Mathis et al., 2018). To train DeepLabCut, 769 total frames were extracted from 29 developmental movies that contained aggregates and/or developmental swarms, which were manually labeled in every frame in which they were present. Using the labeled frames, the DeepLabCut neural network was trained using a 50-layer residual network (He et al., 2016) on Google Colaboratory (hardware accelerator: GPU) for 340,000 iterations (p-cutoff = 0.1). The trained neural network possessed a training and test error of 1.62 and of 6.66 pixels, respectively. To track movement of aggregates and swarms, 15 videos for each strain (3 biological replicates each with 5 technical replicates) from 25 to 72 h of development were analyzed with the trained neural network. The predicted labels (likelihood > p-cutoff) for each video were then manually processed and any spurious labels were removed. To track mobility of individual swarms and aggregates, videos were cropped to contain only a single aggregate and/or swarm. Swarms and aggregates were only analyzed for mobility if they remained within the frame for the entirety of the recording. To track mobility in an entire well, developmental videos (640 × 510 pixels) were initially cropped into 20 smaller videos (160 × 102 pixels) with ImageJ (Schindelin et al., 2012). Each cropped video was analyzed with the trained neural network as stated previously. Labels from individual videos were then manually stitched together. Displacement between two time points with coordinates (x_i_, y_i_) and (x_f_, y_f_) was calculated as [((x_f_ - x_i_)^2^ + (y_f_ - y_i_)^2^)^−2^ × (1661.5 μm/1280 pixels)]. Total swarm displacement was calculated as the sum of all displacements across all time points. Speed of mobility between two time points (t_i_ and t_f_) was calculated as [displacement/((t_f_ – t_i_) × 60 min/h)].

### 2.7 Confocal microscopy

For analysis of single cell *mrpC* expression, *M. xanthus* strains bearing P_van_-*mNeonGreen* and either P_WT_-*mCherry* (PH1373) or P_MUT_-*mCherry* (PH1374) were diluted 1:19 with an unlabeled wild type strain (DZ2) and induced to develop under submerged culture conditions using 2.1 mL diluted cells to seed ibiTreated μ-dishes^35 mm, high^ (Ibidi). Developing cultures were imaged using a Leica TCS SP8 inverted confocal microscope with a 63x objective. Brightfield images were taken with a gain of 230 V and 0.0% offset. mNeonGreen fluorescence was examined using a 488 nm wavelength laser (5% power) for excitation, a 500–540 nm emission spectra, 800 V gain, and 0.0% offset. mCherry fluorescence was examined using a 552 nm wavelength laser (5% power) for excitation, a 585–630 nm emission spectra, 650 V gain, and 0.0% offset. For each replicate of 24 h pre-aggregating cells and 48 h peripheral rods, five images were taken from throughout the plate for each strain (line average: 8, resolution: 1,024 × 1,024). For analysis of the 48 h fruiting bodies, z-stacks of three fruiting bodies were taken for each strain. Each fruiting body was imaged from the base to the interior of the fruiting body (21–37 images; step size: 1 μm, line average: 4, resolution: 1,024 × 1,024). Data were compiled from three independent biological replicates.

Images were subsequently analyzed in ImageJ (Schindelin et al., 2012). For 48 h aggregated populations, images were initially cropped to include only the fruiting body. ROIs were created by thresholding the images from the mNeonGreen channel to contain only pixels that were above the intensity of the unlabeled background strain (pixel threshold: 45), then analyzing particles with area > 0.5 μm^2^ and circularity of 0.0–1.0. ROIs were then transferred to the mCherry channel images and the integrated density was measured. The red-green (RG) ratios were plotted and points identified as outliers by Grubb’s test (*p* < 0.5) were removed. The coefficient of variation (CV) for each biological replicated was calculated by dividing the standard deviation by the mean. Similar RG ratios and CV values were obtained from random single z-layer images indicating no significant differences in *mrpC* reporter expression were observed in different layers of cells.

For analysis of MrpC-mNeonGreen production in developing cells, *M. xanthus* strain PH1375 was induced to develop as above. Prior to imaging, FM 4-64 was added to 5 μg ml^−1^ final concentration and incubated at 32°C for 60 min (modified from Hoang et al., 2021). At the designated time points, developing cultures were imaged as above, except mNeonGreen fluorescence was recorded with 500 V gain. FM 4-64 fluorescence was detected using 722–762 nm emission spectra and 650 V gain. For analysis of peripheral rod populations, five images for each replicate were taken throughout the plate (line average: 8, resolution: 1,024 × 1,024) and ROIs (identified based on membrane stain) were drawn for 20 rod shaped cells and 20 circular cells representing average sizes. For analysis of aggregated cell populations, 1 μm z-stacks of three fruiting bodies or developmental swarms were recorded. One layer in the z-stack was randomly chosen and ROIs were drawn around 40 circular cells within the fruiting body or 40 rod-shaped cells in the swarms each of which was fully in-plane in the image. The mean fluorescence for each ROI in the mNeonGreen channel was measured and plotted.

To account for the relative proportion of spheres and rods in each peripheral rod population, the number of rod-shaped and circular cells were counted in 30.84 × 30.84 pixel ROI (24 h) or 60.07 × 60.07 pixel ROI (30 and 48 h) and a proportional equivalent of random cells or spheres were chosen for plotting. All images were analyzed with the Leica Application Suite X (LasX) histogram tool.

### 2.8 Cell lysate preparation and immunoblot analysis

Cell lysates were generated from strains developed under submerged culture using 16 mL diluted cells seeded in 100-mm petri dishes. At the indicated time points, the overlay buffer was removed, the cell layer was resuspended in 1.5 mL ice-cold MMC. Cells were pelleted (17,000 × g, 5 min, 4°C), and pellets were stored at −20°C until further use. 1 mL 13% ice-cold trichloroacetic acid (TCA) and 0.1 mm zirconia/silca beads were added to each pellet and subject bead beating with a FastPrep-24 tissue homogenizer (MP Biomedical) at 6.5 m/s for 45 s at 4°C, six times with 2 min incubation on ice between rounds. Samples were then incubated on ice for at least 15 min. Protein was pelleted as above, washed with 1.0 mL ice-cold Tris buffer [100 mM Tris–HCl (pH 8.0)], then resuspended in 50 μL Tris buffer and 150 μL clear LSB (Schramm et al., 2012), heated at 95°C for 5 min, then stored at −20°C until further use. Protein concentration was determined by Pierce BCA protein assay kit (Thermo Fisher Scientific), samples were diluted to 0.87 μg μl^−1^ in 2 × LSB, and 10 μg protein resolved by denaturing polyacrylamide (10%) gel electrophoresis and transferred (semi-dry) to polyvinylidene difluoride (PVDF) membrane. Membranes were probed with rabbit polyclonal anti-MrpC (1:1,000) or anti-mNeonGreen (1:1,000) (Cell Signaling Technologies), and anti-rabbit IgG secondary antibodies conjugated to horseradish peroxidase (HRP) at 1:20,000 or 1:5,000, respectively. Signal was detected with enhanced chemiluminescence substrate followed by exposure to autoradiography film.

## 3 Results

### 3.1 Disruption of MrpC binding sites in the *mrpC* promoter region produces increased *mrpC* expression consistent with perturbed negative autoregulation

**Figure 1:**
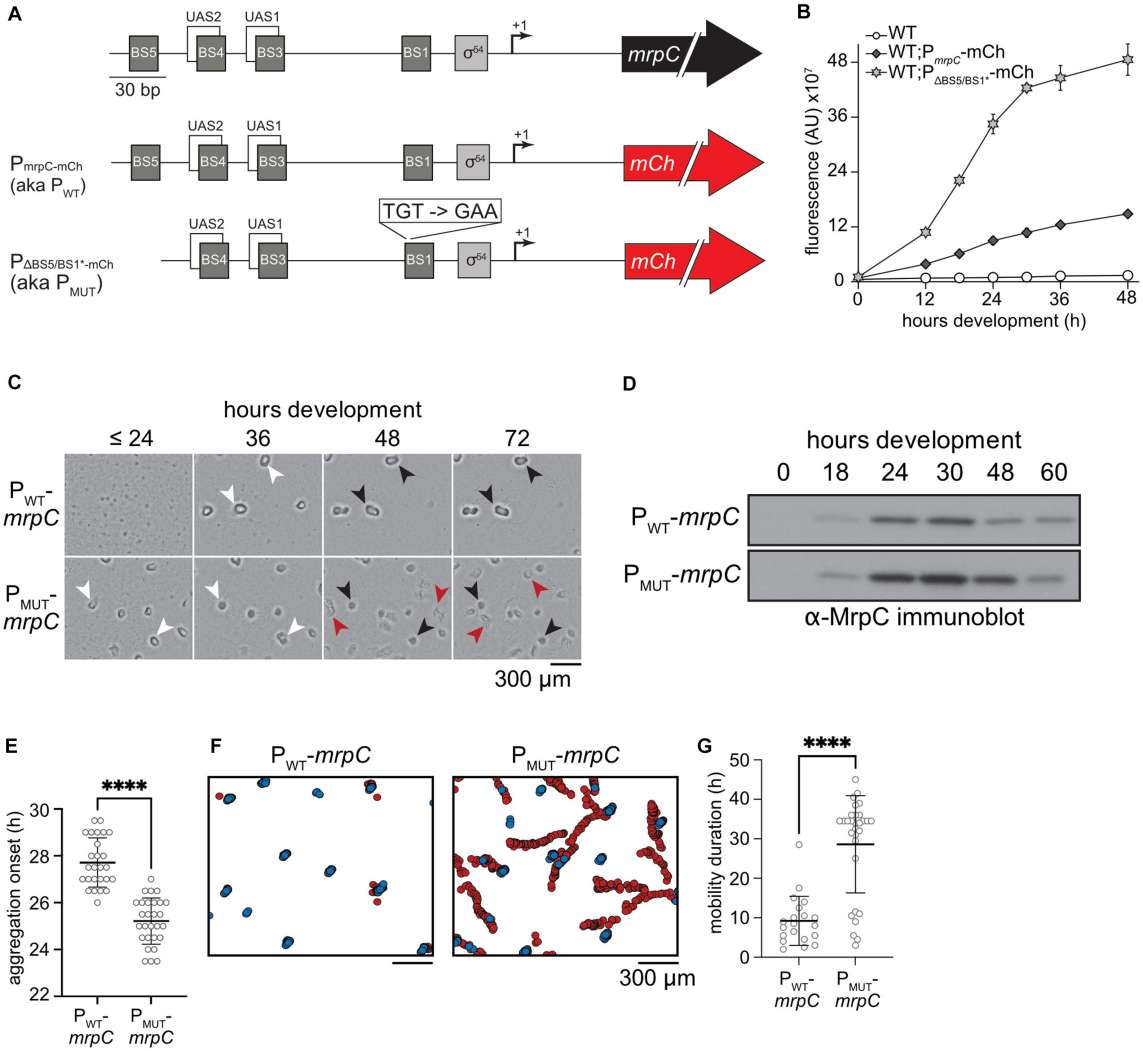
Disruption of MrpC binding sites 1 and 5 perturbs MrpC negative autoregulation (NAR) and leads to asynchronous development. **(A)** Top: Schematic of the *mrpC* promoter region. BS: Sequences to which MrpC directly binds; UAS: upstream activating sequences to which MrpB directly binds; σ^54^: putative sigma^54^-dependent promoter; +1: transcriptional start site; black arrow: *mrpC* gene. Middle: Schematic of the reporter construct used in **(B)**. P_mrpC_-mCh: the wild type *mrpC* promoter region was fused to the mCherry fluorescence reporter gene (red arrow). Bottom: Reporter with deletion of BS5 and disruption of BS1 in the *mrpC* promoter. **(B)** *mrpC* expression is increased when binding sites (BS) 5 and 1 are disrupted. Analysis of *mrpC* expression with an mCherry reporter expressed from the wild type *mrpC* (P*_mrpC_*-*mCh*) or mutant MrpC binding sites 1 and 5 (P_ΔBS5/BS1*_-*mCh mrpC*) promoters. Reporters were integrated into wild type cells and mCherry fluorescence was recorded during development under submerged culture. Data plotted is the average and associated standard deviation of three independent biological replicates. **(C)** Disruption of MrpC BS 5 and 1 caused early aggregation and disrupted fruiting bodies. The developmental phenotype observed from Δ*mrpC* cells expressing *mrpC* from its wild type- (P_WT_-*mrpC*) or P_ΔBS5/BS1*-_ (P_MUT_-*mrpC*) promoters recorded at the hours post-starvation indicated; >24 indicates images recorded at 0, 12, 18, and 24 h of development were indistinguishable. White arrows: aggregates of ~10^5^ cells; black arrows: immobile fruiting bodies; red arrows: developmental swarms. **(D)** Representative anti-MrpC immunoblot of lysates prepared from P_WT_-*mrpC* or P_MUT_-*mrpC* cells developing under submerged culture. **(E,G)** Distribution of developmental times at which aggregation centers were first observed (aggregation onset; **E**), or durations of time that aggregates (P_WT_-*mrpC*) or developmental swarms (P_MUT_-*mrpC*) traveled (mobility duration; **G**). Mean (bar) and associated standard deviations are indicated. Data was analyzed for statistically significant differences using an unpaired *t* test **(E)** or Mann–Whitney test **(G)**. ****p < 0.0001. Strains were developed under submerged culture in 96 well plates and imaged every 30 min for 72 h Data are compiled from three independent biological replicates. *n* = 26 (P_WT_-*mrpC*) and 28 (P_MUT_-*mrpC*) **(E)**; *n* = 20 (P_WT_-*mrpC*) and 30 (P_MUT_-*mrpC*) **(G)**. **(F)** Identification and tracking of aggregates by a DeepLabCut trained neural network. Stationary aggregates (blue) and developmental swarms (red) identified from movies of cells developing under submerged culture as in **(C)**. Data from one representative assay is shown.

*mrpC* expression is subject to NAR due at least in part to competition between MrpC and its transcriptional activator, MrpB, for overlapping binding sites (McLaughlin et al., 2018) (Figure 1A). MrpC binds to two additional sequences within its promoter (termed BS5 and BS1) (McLaughlin et al., 2018). To examine whether BS5 and BS1 contributed to MrpC NAR, we analyzed *mrpC* reporter constructs bearing either the wild-type promoter or mutant promoters containing substitutions within BS1 (BS1*), a deletion of BS5 (ΔBS5), or both (BS1*ΔBS5). BS1* was generated by substituting the TGT consensus resides to GAA which completely disrupts MrpC binding to BS1 *in vitro* (McLaughlin et al., 2018) (Figure 1A; Supplementary Figure S1B). Each reporter construct was inserted into the Mx8 phage attachment site (*attB*) of wild-type *M. xanthus* strain DZ2, and developmental assays indicated all resulting strains displayed wild-type developmental phenotypes (data not shown). Analysis of the reporters during development demonstrated the ΔBS5 and BS1* mutations resulted in 2.5-fold and 1.8-fold increases in mCherry fluorescence compared to the wild-type reporter at 48 h, respectively (Supplementary Figure S1C). The reporter bearing the BS1* ΔBS5 double disruption yielded a 3.3 fold increase in mCherry fluorescence compared to the wild type parent, and 1.3-fold increase compared to the single ΔBS5 reporters (Figure 1B; Supplementary Figure S1C). No significant mCherry fluorescence was detected from the P_ΔBS5-3_-mCh reporter which lacks the MrpB binding sites UAS1 and 2 as well as MrpC BS3, 4 and 5 (McLaughlin et al., 2018) (Supplementary Figures S1A,C), indicating no other promoter elements contribute to induction of *mrpC* expression. The observation that reporter expression in the Δ*mrpC* strain is dramatically higher early during development (Supplementary Figure S1D) reflects the contribution of MrpC binding to BS3/4 to directly compete with MrpB binding at UAS1/2. MrpC BS3/4 intimately overlap with MrpB UAS1/2 (McLaughlin et al., 2018), and we could not design mutations which would not also be predicted to perturb MrpB-dependent activation of *mrpC* expression.

### 3.2 Mutation of MrpC BS1 and BS5 leads to early aggregation and reduced sporulation

To examine how the MrpC BS1 and BS5 mutations affect *M. xanthus* development, we next generated constructs in which the *mrpC* gene was driven from its wild-type promoter or from the ΔBS5/BS1* promoter. These constructs were integrated in the Δ*mrpC* background at the *attB* site, producing Δ*mrpC attB*::P_WT_-*mrpC* (parent) and Δ*mrpC attB*::P_ΔBS5/BS1*_ -*mrpC* strains (hereafter termed P_WT_-*mrpC* and P_MUT_-*mrpC*, respectively). The phenotype of these two strains was compared to the Δ*mrpC* and wild type background strains induced to develop under submerged culture. In this assay, cells are first allowed to grow into a uniform layer that covers the bottom of the well, and development is induced by replacing growth media with starvation buffer. As expected, the Δ*mrpC* strain failed to produce aggregates, whereas the wild-type and P_WT_-*mrpC* strains produced similar visible aggregation centers between 24 and 36 h that darkened by 72 h post-starvation (Figure 1C; Supplementary Figure S2A), indicating exogenously expressed *mrpC* restores wild type aggregation. In contrast, the P_MUT_-*mrpC* strain produced aggregates at least 6 h earlier than the parent strain (Figure 1C; Supplementary Figure S2A). Furthermore, while the P_MUT_-*mrpC* aggregates appeared similar to the parent at 36 h, they subsequently failed to appropriately darken, and by 48–72 h became more disorganized than the parent and wild-type strains (Figure 1C).

To examine sporulation levels in these strains, heat- and sonication-resistant spores were enumerated at 48, 72, and 120 h. After 120 h of development, the wild type had produced 3.1 ± 0.7 × 10^7^ spores (recorded as 100 ± 23%), while no spores could be detected from Δ*mrpC* mutant (≤0.07% wild type) (Supplementary Figure S2B). The P_WT_-*mrpC* strain produced 70 ± 16% of the wild type levels, suggesting exogenous expression of *mrpC* did not fully complement with respect to sporulation efficiency (Supplementary Figure S2B). The P_MUT_-*mrpC* strain, however, exhibited a striking reduction in sporulation corresponding to 30 ± 17% of the resistant spores produced by the P_WT_-*mrpC* strain at 72 h of development (Supplementary Figure S2B). To determine if there was an inherent defect in the core sporulation program in the P_MUT_-*mrpC* mutant, we examined the number of heat- and sonication-resistant spores produced after artificial chemical induction of sporulation in liquid cultures which bypasses the requirement for aggregation (Dworkin and Gibson, 1964). The P_MUT_-*mrpC* mutant produced a similar number of chemical-induced spores as the wild type, whereas the Δ*mrpC* mutant failed to produce any spores (Supplementary Figure S2C). These results suggested that the inefficient sporulation observed by the P_MUT_-*mrpC* strain during starvation-induced development was not due to failure to execute spore differentiation *per se*.

Finally, to examine how the observed phenotypes correlated with total MrpC levels, we prepared lysates from P_WT_-*mrpC* or P_MUT_-*mrpC* strains at intervals between 0 and 60 h of development and subjected them to anti-MrpC immunoblot. In the P_WT_-*mrpC* strain, MrpC was absent at the onset of development (*t* = 0), increased between 18 and 30 h of development, and then subsequently decreased after the onset of sporulation (Figure 1D; Supplementary Figure S2B). Relative to the P_WT_-*mrpC* strain, levels of MrpC in the P_MUT_-*mrpC* strain were 2-3-fold higher between 18 and 48 h, and eventually decreased to P_WT_-*mrpC* levels by 60 h (Figure 1D; Supplementary Figure S3B). This pattern of MrpC accumulation in the two strains was similar to *mrpC* expression levels when the relative increase in mCherry production was examined by plotting the change in mCherry fluorescence between n and *n* + 1 time points (Supplementary Figure S3). Consistent with previous observations (Higgs et al., 2008; Schramm et al., 2012), elevated MrpC levels at 24 h likely explained the early aggregation onset observed in the P_MUT_-*mrpC* strain (Figure 1C). However, the reduced sporulation efficiency observed by this strain was surprising given MrpC levels were similar at 60 h of development when sporulation levels were reduced compared to the parent (Supplementary Figure S2B). Together, these results suggested that perturbing the binding of MrpC in its promoter region, and likely interfering in MrpC NAR, resulted in an uncoupling between completion of aggregation and induction of sporulation.

### 3.3 Perturbation of MrpC NAR leads to asynchronous development

Movies of *M. xanthus* development have demonstrated that prior to the onset of sporulation, aggregates are surprisingly dynamic (Curtis et al., 2007; Xie et al., 2011; Zhang et al., 2011; Bahar et al., 2014; Glaser and Higgs, 2019). Initial aggregates often dissolve or coalesce, and even mature aggregates can be mobile prior to transition to stationary spore-filled fruiting bodies (Glaser and Higgs, 2019). To examine how MrpC NAR affected these transient behaviors, the wild type, P_WT_-*mrpC* (Supplementary Movie S1), and P_MUT_-*mrpC* (Supplementary Movie S2) strains were induced to develop under submerged culture conditions, imaged every 30 min from 0 to 72 h in an automated plate reader, and the images assembled into movies. For each movie, the time of aggregation onset, the number of initial vs. mature aggregates, and the duration of mature aggregate mobility was recorded. These analyzes demonstrated that aggregation onset in the P_MUT_-*mrpC* strain assays was 3 h earlier than the wild type and P_WT_-*mrpC* strains (25 ± 1 vs. 28 ± 1 and 28 ± 1 h post-starvation, respectively) (Figure 1E; Supplementary Figure S2D; Supplementary Table S3).

Remarkable differences in the behavior of mature aggregates between the strains was detected. In the wild type strain, very few mobile aggregates were observed (5 ± 12%) and the duration of mobility was short (2 ± 1 h) (Supplementary Table S3). In the P_WT_-*mrpC* strain, 40 ± 30% of the aggregates were mobile for 7 ± 3 h (Figures 1F,G; Supplementary Table S3; Supplementary Movie S1). As the endogenous *mrpC* promoter region is still intact in the Δ*mrpC* background, we speculated that the differences between the wild type and P_WT_-*mrpC* (i.e., Δ*mrpC attB*::P_WT_-*mrpC*) strains may result from two copies of the *mrpC* promoter region, which may dilute the NAR activity of the available MrpC. Remarkably, however, in 80 ± 20% of the aggregates produced in the P_MUT_-*mrpC* strain, a large proportion of cells suddenly exited one side of the aggregate, leaving other cells behind as darkened shallow fruiting bodies (Supplementary Movie S2). The cells that exited the aggregate collectively migrated throughout the plate; we referred to these cells as a developmental swarm. Most of these swarms (70%; 19/27) were still actively moving by the end of the filming period at 72 h. These observations explained both the disorganized appearance of the late aggregates (Figure 1C) and the reduced sporulation efficiencies (Supplementary Figure S2B) observed in the P_MUT_-*mrpC* strain.

On average, all three strains produced similar maximum numbers of initial aggregates (between 6 and 8) and final fruiting bodies (between 5 and 6) (Supplementary Table S3). However, in the P_MUT_-*mrpC* strain, the overall number of aggregates that transitioned into fruiting bodies (60 ± 10%) was significantly reduced (*p* = 0.031; *t*-test) compared to the wild type and P_WT_-*mrpC* strains (80 ± 16% and 80 ± 19%, respectively) (Supplementary Table S3; Supplementary Figure S4B). Interestingly, although aggregation onset in the P_MUT_-*mrpC* cells was 3 h earlier than the other strains, there was no significant difference in the time at which fully formed aggregates began to move (mobility onset) in the P_MUT_-*mrpC* (37 ± 4 h) relative to the P_WT_-*mrpC* cells (37 ± 4 h) (Supplementary Table S3; Supplementary Figure S4A). Thus, the interval between formation of aggregates and onset of aggregate mobility in the P_WT_-*mrpC* and P_MUT_-*mrpC* was 7 ± 3, and 11 ± 3 h, respectively.

To better compare the characteristics of the individual entities produced by the P_MUT_-*mrpC* and P_WT_-*mrpC* mobile strains, we employed the DeepLabCut deep convolutional neural network (Mathis et al., 2018) to analyze developing strains. The neural network was trained on manually labeled P_WT_-*mrpC* or P_MUT_-*mrpC* developmental images with a 50-layer residual network (ResNet-50) for 340,000 iterations resulting in a training and test error of 1.62 and 6.66 pixels, respectively (Becskei and Serrano, 2000; He et al., 2016). The trained neural network was then used to analyze 15 videos of each strain (3 independent biological replicates each with 5 technical replicates). First, the neural network assigned non-mobile aggregates a median speed of 0.22 μm/min (IQR: 0.12–0.40 μm/min), which was attributed to an artifact from slight shifts in the position of the automated plate reader camera between time points (data not shown). In the P_WT_-*mrpC* strain, the mobile aggregates traveled at an average speed of 0.3 μm/min (Supplementary Figure S4F), and movement was primarily radial (Figure 1F, Supplementary Figure S4C) such that the average net displacement was within two aggregate diameters of the starting point (Figure 1F, Supplementary Figure S4E). In the P_MUT_-*mrpC* strain, developmental swarms traveled at an average speed of 0.6 μm/min (Supplementary Figure S4F) and their movement involved long runs, sharp turns, and/or repeated reversals (Figure 1F, Supplementary Figure S4D); the average total displacement was 1,500 ± 400 μm (Supplementary Figure S4E). Unlike with the P_WT_-*mrpC* strain, almost all mobile aggregates left a shallow immobile fruiting body behind (Figures 1C,F). Thus, developmental swarms displayed speed and trajectory characteristics that were significantly different from the P_WT_-*mrpC* mobile aggregates, suggesting they did not result from simply increasing the duration of the parent aggregate mobility phase.

### 3.4 MrpC NAR dampens cell-to-cell variability in *mrpC* expression

Theoretical and experimental analyzes have suggested that NAR motifs function to decrease cell-to-cell variability in gene expression, ensuring that expression is homogenous within a population (Becskei and Serrano, 2000). Since the P_MUT_-*mrpC* strain appeared to produce subpopulations of cells in different developmental states (i.e., developmental swarms and stationary fruiting bodies; Figure 1F), we hypothesized that MrpC-mediated NAR may be functioning to limit heterogeneity of *mrpC* expression thus ensuring a coordinated developmental process.

To examine population variation in *mrpC* expression, we measured mCherry production from individual wild-type cells bearing the P_WT_-mCh or P_MUT_-mCh reporter during *in situ* development under submerged culture. For normalization purposes, these cells also contained a construct that expressed mNeonGreen from an inducible promoter integrated at a second genomic site (1.38 kb::P_vanillate_-*mNeonGreen*). These double labeled strains were each diluted 1:19 into a markerless wild type, and single cell fluorescence was recorded using confocal laser scanning microscopy (CLSM). For each cell, mCherry fluorescence was normalized to mNeonGreen fluorescence, and the distribution of red-to-green (RG) ratios from individual cells from three independent biological experiments was plotted. Variability in single-cell RG ratios among the population was calculated using the coefficient of variation (CV).

**Figure 2:**
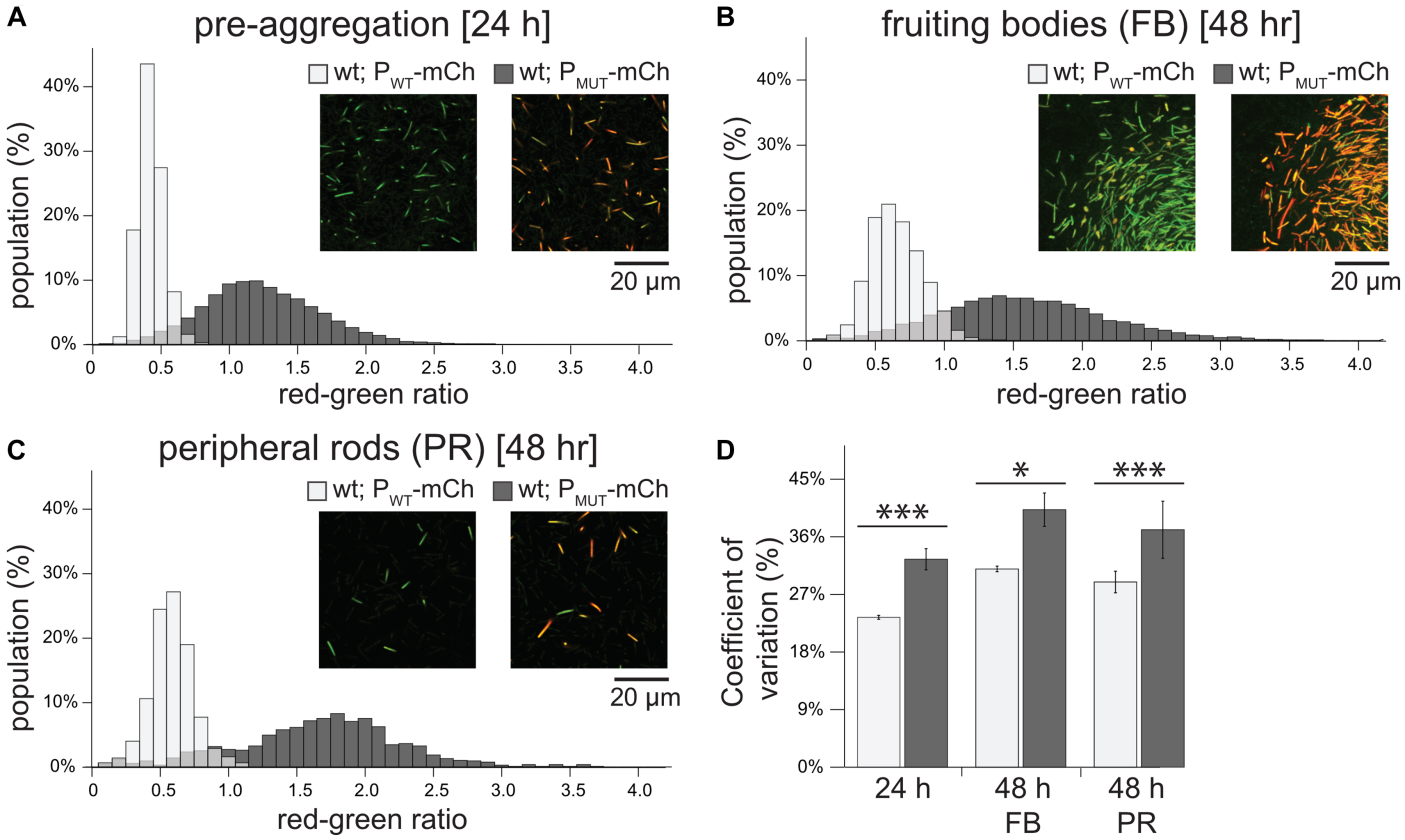
Perturbation of MrpC NAR increases the level and population-wide variability of *mrpC* expression. **(A–C)** Histogram of *mrpC* reporter expression in pre-aggregation **(A)**, fruiting body **(B)**, or peripheral rod populations **(C)**. Wild-type cells expressing mCherry from either the wild-type *mrpC* promoter (P_*mrpC*_-*mCh*; P_WT_-*mCh*) or perturbed NAR *mrpC* promoter (P_BS5/BS1*_-*mCh*; P_MUT_-*mCh*), and mNeonGreen from a constitutive promoter (P_vanillate_-*mNG*). Strains were developed under submerged culture and imaged by confocal microscopy. mCherry (red) and mNeonGreen fluorescence was measured for individual cells and the red-green ratio calculated. Ratios were binned and the percent of the population with the indicated red-green ratio displayed by histogram. For the P_WT_-mCh strain, *n* = 8,064 **(A)**, 14,298 **(B)**, and 933 **(C)** cells. For the P_MUT_-mCh strain, *n* = 5,835 **(A)**, 11,198 **(B)**, and 850 **(C)** cells. Representative images showing mCherry and mNeonGreen fluorescence overlayed from each population in the indicated strains is shown. Note: the edge of a fruiting body is shown in **(B)**. **(D)** Average coefficient of variation and associated standard deviation was calculated from three independent biological replicates. Light gray: P_WT_-*mCh*; dark gray: P_MUT_-*mCh*. Data was analyzed for statistically significant differences using an unequal variances *t*-test. *p < 0.05; ***p < 0.001.

In pre-aggregating cells (24 h development) with the wild type reporter, a mean RG ratio of 0.38 ± 0.09 was observed with a narrow distribution in values (Figure 2A) that corresponded to a CV of 23.4 ± 0.3% (Figure 2D). Cells with the P_MUT_-mCh reporter displayed both 3-fold increased mean reporter expression (RG ratio of 1.2 ± 0.4) and a significantly increased spread in distribution of expression (CV 32 ± 2%) (Figures 2A,D). Similarly, in fruiting bodies, cells bearing the P_MUT_-mCh reporter displayed 2.5- and 1.3-fold increased mean RG ratio and CV compared to the P_WT_-mCh fruiting body cells, respectively (Figures 2B,D). This trend was also observed in the peripheral rods (mean RG ratio and CV of 3- and 1.3-fold higher than the P_WT_-mCh reporter, respectively). These results indicated that MrpC-mediated NAR may function to not only limit the level of expression, but also to limit the cell-to-cell variability in expression. Additionally, these results suggested the increase in variability was not subpopulation specific, indicating that MrpC-mediated NAR was functioning throughout the entire population to limit expression heterogeneity.

### 3.5 Developmental swarms possess an intermediate level of MrpC

**Figure 3:**
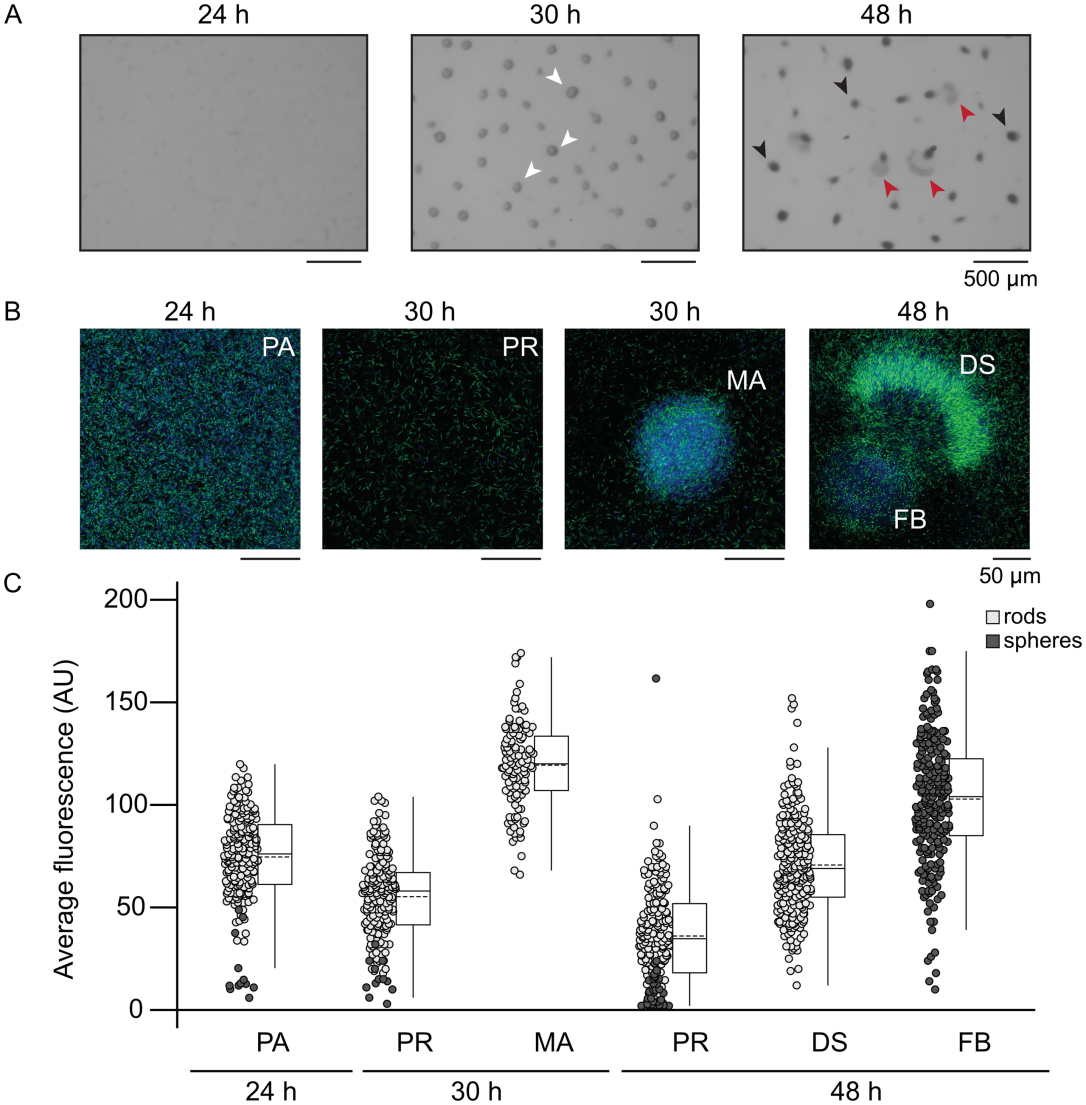
Developmental swarms correlate with intermediate MrpC production. **(A)** Developmental phenotype images of the Δ*mrpC* strain expressing *mrpC-mNG* from the wild type *mrpC* promoter integrated at an exogenous locus (P_WT_-*mrpC*-*mNG*). **(B)** Fluorescent imaging of P_WT_-*mrpC*-*mNG* cells in the indicated stages. Development was induced under submerged culture conditions for the indicated times, treated with FM4-64 membrane stain, and imaged by confocal microscopy. Fluorescence captured from the membrane strain and mNeonGreen is colored blue and green, respectively. **(A,B)** Pre-aggregation cells (PA), mature aggregates (MA, white arrows), developmental swarms (DS, red arrows), and fruiting bodies (FB, black arrows) are indicated. **(C)** Average mNeonGreen fluorescence recorded from individual rod shaped cells (light gray circles) or spherical cells (dark gray circles) in each population. Data distribution is indicated to the right by box plots. Boxes: first quartile-third quartile; whiskers: minimum and maximum; solid line: median; dashed line: mean. Outliers show as distinct dots. Regions of interest (ROIs) were identified based on the membrane stain and the average mNeonGreen fluorescence within the ROI was measured. Results from two independent biological replicates are shown. *n* = 200 cells for PA, 30 h PR and 48 h PR populations. *n* = 240 cells for 30 h MA, 48 h FB, and 48 h DS populations. Circles in the pre-aggregate and peripheral rod populations likely corresponded to end-on cells, rather than spores. Statistical analysis (two-sample unequal variances *t*-test) indicated all populations displayed significantly different average fluorescence intensities (*p* < 0.001), except 48 h DS and 24 h PA (*p* = 0.08).

Thus far, we observed that disruption of MrpC BS1/5 led to uncoordinated behavior and increased variability in *mrpC* expression within the population. To examine the MrpC levels in individual cells in the distinct subpopulations, we set out to generate strains producing MrpC fused to a fluorescent marker. Attempts to generate strains producing MrpC with fluorescent proteins or small fluorescent tags fused to either the amino- or carboxy-terminus, expressed either from the endogenous *mrpC* locus or from exogenous sites, resulted in strong developmental phenotypes and/or partial release of MrpC from the fusion proteins (data not shown). One interesting exception was a strain bearing a carboxy-terminal mNeonGreen fusion to MrpC expressed from the native *mrpC* promoter in the Δ*mrpC* background (Δ*mrpC attB*::P_WT_-*mrpC-mNeonGreen*; hereafter termed *mrpC*-*mNG*). Surprisingly, this strain exhibited a similar developmental swarm phenotype to that of the P_MUT_-*mrpC* (Supplementary Figure S5A, Supplementary Movie S3) although aggregation onset was 6 h earlier in the *mrpC*-*mNG* strain than in the P_MUT_-*mrpC* strain, and there was more variability in the number of mobile aggregates between biological replicates (Supplementary Table S3). These results strongly suggested that fusion of mNeonGreen to the C-terminus of MrpC was interfering in efficient NAR, perhaps because the mNG fusion interferes with cooperative multimeric MrpC interactions required for efficient exclusion of MrpB from the *mrpC* promoter region (McLaughlin et al., 2018). Examination of developing *mrpC*-*mNG* cells by fluorescence microscopy demonstrated that fluorescence was detected primarily in the center of the cell (Supplementary Figure S5C) likely colocalized with the nucleoid, consistent with the role of MrpC as a global transcriptional regulator. A similar localization was also observed by anti-MrpC immunofluorescence in wild-type developing cells (V. Bhardwaj and P. I. Higgs, unpublished results). Finally, immunoblot analyzes of the developmental *mrpC*-*mNG* lysates demonstrated no untagged MrpC could be by detected by polyclonal anti-MrpC immunoblot, indicating MrpC-mNG (predicted molecular mass 54 kDa) was the sole version of MrpC in this strain (Supplementary Figure S5B). However, anti-mNG antibodies detected bands corresponding to the 54 kDa MrpC-mNG fusion protein and a 27 kDa mNG monomer (Supplementary Figure S5B). We speculate mNeonGreen was released as a result of normal MrpC turnover (Schramm et al., 2012); importantly, this mNeonGreen should not be localized over the nucleoid and was likely detected as diffuse signal in the cells. Regardless, mNeonGreen fluorescence indicated the level of MrpC that was (at one point) produced. Therefore, we took advantage of the *mrpC*-*mNG* strain to examine the relative accumulation of MrpC in developing cells in the pre-aggregation, developmental swarm, sporulating fruiting body, and peripheral rod populations *in situ*. For this purpose, the *mrpC*-*mNG* strain was induced to develop for 24, 30, or 48 h and stained with a membrane dye (FM4-64) for 60 min prior to imaging by CLSM. At the pre-aggregation stage (24 h post-starvation), we observed randomly aligned rod-shaped cells with a mean per cell mNeonGreen fluorescence of 80 ± 20 arbitrary units (AU) (Figures 3A–C). By 30 h, the population divided to produce cells within symmetric round aggregates and peripheral rods remaining between the aggregates with mean per cell fluorescence of 120 ± 20 AU and 60 ± 20 AU, respectively. The observation that mNG fluorescence levels in these two populations correlated with relative levels of MrpC previously observed in peripheral rod populations vs. aggregate populations in *M. xanthus* strain DZ2 (Lee et al., 2012) strongly suggests that mNG fluorescence was also useful for estimating relative levels of MrpC in the developmental swarms. Developmental swarms observed at 48 h contained per cell fluorescence values (70 ± 20 AU) between those observed for cells in the residual fruiting bodies (100 ± 30) and peripheral rods (40 ± 20) (Figures 3A–C). The developmental swarms consisted almost exclusively of rods aligned with their long axis perpendicular to the moving front of the swarm, whereas the fruiting bodies consisted of rods aligned tangential to the edge of the fruiting body or spherical shaped spores (Supplementary Figure S5D). At both 30 and 48 h, the peripheral rods were randomly aligned (Supplementary Figure S5D). These observations suggested that developmental swarms arise from cells locked into an intermediate MrpC level. We speculate these intermediate MrpC levels promote cell movement, whereas cells that attained a higher level of MrpC had reduced cell movement and triggered differentiation into non-motile spores forming immobile fruiting bodies.

## 4 Discussion

MrpC is a key developmental transcriptional regulator of the multicellular developmental program in *M. xanthus.* MrpC accumulation is key to dictate onset of stages (i.e., aggregation and sporulation) within the developmental program, but also likely determines distinct cell fates within the program (Figure 3; Lee et al., 2012). MrpC is subject to negative autoregulation and its promoter region contains at least four MrpC binding sites (McLaughlin et al., 2018). Disruption of either binding site 1 or 5 led to increased *mrpC* expression (Figure 1; Supplementary Figure S1), and disruption of both produced a nearly additive effect. The arrangement of BS1 suggests that MrpC dimers sterically hinder binding of the sigma^54^ factor to its promoter, consistent with DNase foot printing analyzes (Nariya and Inouye, 2006). MrpC binding at BS5 may additionally interfere with MrpB binding to UAS1. We further hypothesize that MrpC bound at BS5 and BS1 may interact to stabilize a DNA loop that further impedes MrpB access to UAS1/2, because deletion of an unusual 25 amino acid N-terminal extension in MrpC has the same effect on the *mrpC* reporter as the Δ*mrpC* deletion strain, but is completely competent for binding at any of BS 1, 3, 4, and 5 *in vitro* (McLaughlin et al., 2018). Thus, the BS1/BS5 mutations likely perturbed MrpC NAR. Consistently, a known attribute of NAR is to constrain variation in gene expression (due to noise reduction), and the BS1/BS5 mutation lead to increased cell-to-cell variation in *mrpC* expression (Figure 2). However, we could not address other known attributes of NAR that have been observed in synthetic systems because we did not isolate MrpC from its additional feedback circuits (examples described below).

One phenotypic consequence of the BS1/BS5 mutations was production of aggregation centers earlier than the parent (Figures 1C,E), likely because MrpC accumulated earlier in this strain (Figure 1D). MrpC levels exhibit similar accumulation patterns in the absence of the Esp signaling system, which induces proteolytic turnover of MrpC (Cho and Zusman, 1999; Schramm et al., 2012). *esp* mutants likewise display early aggregation onset but, in contrast to the P_MUT_-*mrpC* strain, also result in early and increased sporulation efficiency (Cho and Zusman, 1999; Schramm et al., 2012). The P_MUT_-*mrpC* strain displayed significantly reduced sporulation efficiency because some cells abruptly exited the aggregation centers and remained locked in a swarming state (Figure 1F; Supplementary Figure S2). This phenomenon can be attributed to increased variability in *mrpC* expression (Figure 2) rather than just increased levels *per se*, because the *esp* mutant does not produce developmental swarms (Schramm et al., 2012) (Supplementary Movie S4). Instead, the *esp* strain skipped the aggregate mobility phase and transitioned into darkened immobile fruiting bodies earlier than the wild type (Supplementary Movie S4).

**Figure 4:**
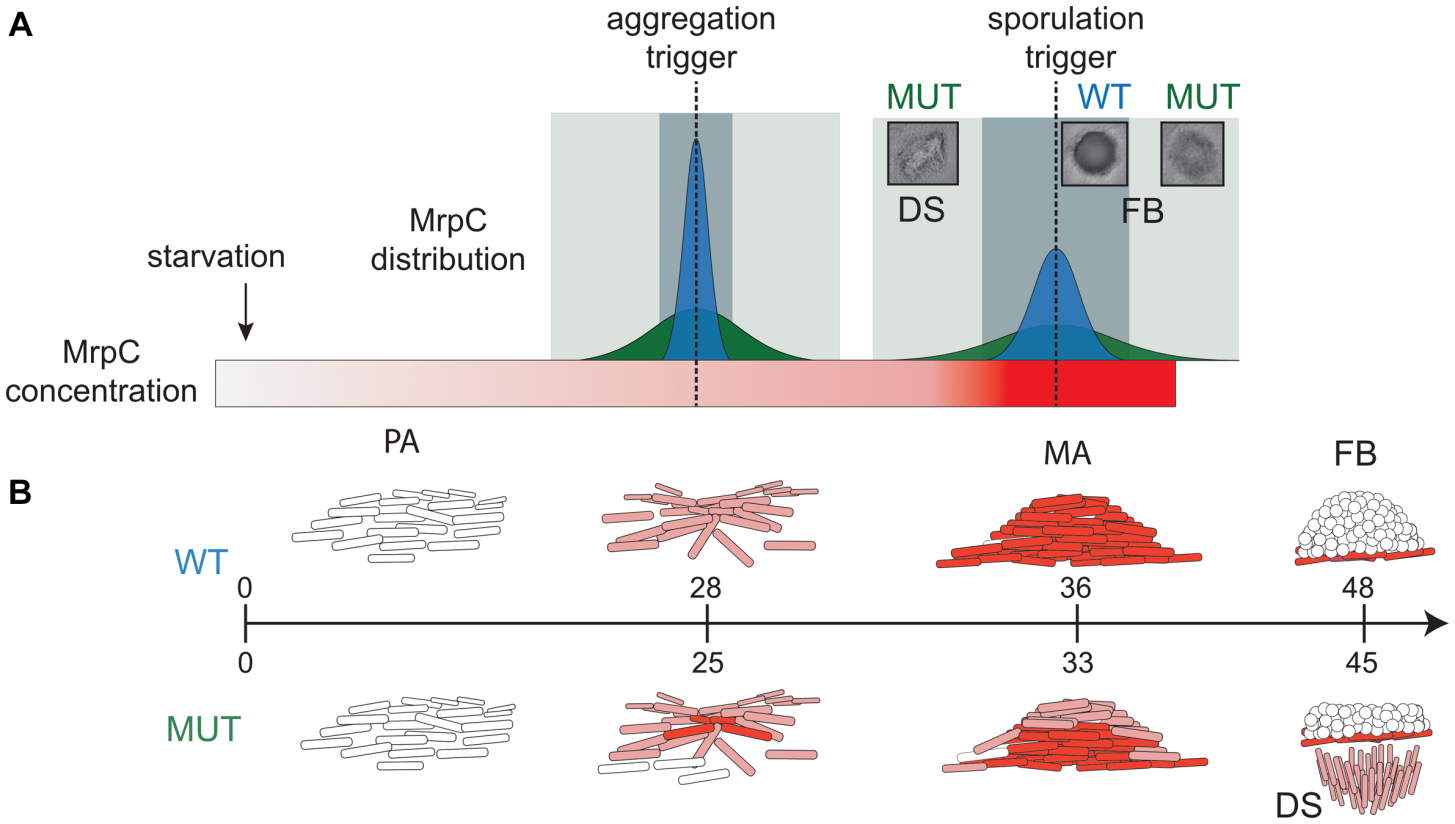
Model for role of NAR in synchronized progression through developmental phases. **(A)** Distributions of MrpC in the wild-type (dark blue peak) and perturbed MrpC NAR (dark green peak) strains at trigger points (dotted lines) for aggregation and sporulation. After the onset of starvation, MrpC levels (red gradient rectangle) steadily increase. With appropriate NAR, the distribution of MrpC at the trigger thresholds is constrained which promotes synchronized transition of the population to aggregation and then sporulation phases. The window for population transition to the next stage is short (light blue rectangle). When NAR is perturbed, the distribution of MrpC at the trigger thresholds is broad and cells can contain different threshold levels of MrpC. The window for population transition to the next stage is lengthened (light green rectangle) causing some cells to swarm away from fruiting bodies. **(B)** Schematic of *M. xanthus* developmental stages and associated MrpC levels in individual cells in the wild type (WT; top) and perturbed MrpC NAR mutant (MUT; bottom). WT: Cells (white rods) in the pre-aggregation (PA) stage contain little MrpC. Accumulation of MrpC (light red) triggers aggregation, and cells move into aggregation centers (mounds of cells). Continued accumulation of MrpC (dark red) in mature aggregates (MA) triggers sporulation (white circles) forming fruiting bodies (FB). Mature committed spores contain little MrpC. MUT: Broadened distribution of MrpC results in multiple stages of development at the same time. MAs contain some cells that reached the sporulation threshold. Cells which did not reach the sporulation threshold exit the aggregate as developmental swarms (DS), leaving residual fruiting bodies in place. Peripheral rods are not shown. Aggregation onset is induced on average 3 h earlier than in the wild type.

The increased cell-to-cell variability in *mrpC* expression likely produced a population that simultaneously contains a mixture of cells at different MrpC thresholds (i.e., pre-aggregation, aggregation, or sporulation) (Figure 4). For example, at 30 h of development, the population consisted of cells that had already accumulated higher levels of MrpC and were already in mature aggregates (Figure 3), as well as cells which had not yet accumulated much MrpC and had likely not yet aggregated into these centers. By 48 h, some of these cells had already reached the level of MrpC required to commit to sporulation and thus remained in stationary fruiting bodies (Figure 3). Those at the lower end were in the aggregation phase and collectively exited the aggregation center as a swarm. With the wild-type *mrpC* promoter, MrpC levels are more homogeneous within a given time frame, and the population undergoes a quick, collective transition from aggregation to sporulation (Figure 4). Thus, MrpC negative autoregulation appears to be important in maintaining population synchrony during the *M. xanthus* multicellular developmental program.

The swarms that exited the mounds exhibited aberrant behavior because they left the mature aggregates and continued to migrate as a group until at least 72 h post-starvation. How could the P_MUT_-*mrpC* lead to this novel behavior? One possibility is that cells inside an aggregation center are normally subject to an additional positive feedback loop which is necessary to reinforce transition to sporulation. For example, C-signaling, a contact-dependent signaling system is highly active in aggregated cells and particular in cells transitioning to spores (Sogaard-Andersen and Kaiser, 1996; Hoang et al., 2021), but is presumably inactivated once the cells differentiate into spores. In the P_MUT_-*mrpC* strain, developmental swarms appear to arise from cells that expressed lower levels of MrpC and arrived at the mature aggregate after the other cells had already sporulated. These late-to-arrive cells may have therefore missed the positive feedback loop necessary to increase MrpC levels to the sporulation threshold (Supplementary Figure S6A), and are instead stuck at intermediate MrpC levels which stimulate aggregation. A second (not mutually exclusive) possibility is that an oscillation motif is triggered in the developmental swarms such that MrpC levels never reach the sporulation threshold but never fall below the aggregation threshold. It is known that oscillations can be generated through a network motif comprised of a composite negative feedback loop coupled to an additional positive feedback loop (Alon, 2007; Stricker et al., 2008). Interestingly, such a motif can be identified for MrpC (Supplementary Figure S6B): MrpC induces expression of *esp* (S. Kasto, A. Schramm and P. I. Higgs, unpublished results), and Esp indirectly induces degradation of MrpC via proteolysis (Schramm et al., 2012). MrpB induces expression of *mrpC*, and MrpC positively regulates expression of *mrpB* (Sun and Shi, 2001a) (C. Mataczynski and P.I. Higgs, unpublished results). Another possibility is that the propagation of these swarms could arise from changes in mechanical forces that govern cell swarms, such as local changes in surfactant or cell–cell adhesion properties (Guzman-Herrera et al., 2020; Islam et al., 2020; Saïdi et al., 2021). The observation that strains that produce the developmental swarms initially produce relatively normal aggregates, is consistent with models suggesting initial aggregates (cell layers) form as a result of physical forces governing self-organization, such as Oswald ripening or active nematic liquid crystal formation (Bahar et al., 2014; Copenhagen et al., 2021). The role of distinct levels of MrpC in strain DZ2 may then be to control the proportions of cells in distinct fates and stabilize transition of aggregates into mature fruiting bodies.

During development in *M. xanthus*, groups of cells organize into a defined pattern (i.e., fruiting bodies) for a designated function (protection and/or collective dispersal), making the developing population akin to a specialized bacterial tissue. While multicellular tissue formation in *M. xanthus* is relatively simple compared to that of higher eukaryotic organisms, many of the same basic regulatory principles still apply. Cells must synchronously progress through development in a defined temporal order to produce a functional structure. This principle is exemplified by the regulation of gastrulation in *Drosophila melanogaster* embryogenesis. Invagination (coordinated cell movement) during gastrulation is largely coordinated by the key regulator, *snail* (Leptin and Grunewald, 1990). Expression of *snail* displays a significant degree of homogeneity and synchronicity, which is crucial for its function (Boettiger and Levine, 2013; Lagha et al., 2013). If synchronicity is perturbed, then multicellular coordination of invagination becomes defective and the severity of the defect strongly correlates with the level of asynchronicity (Lagha et al., 2013). Intriguingly, Snail is proposed to function as a negative autoregulator, which is thought to promote homogeneity of *snail* expression in the population (Boettiger and Levine, 2013); this provides an additional example where the noise reduction attribute of NAR motifs may promote synchronized responses in a multicellular developmental systems.

Our study also illustrates a mechanism for evolution of emergent behaviors. Developmental swarms, a distinct subpopulation of cells stuck in a collective movement state, arose from mutations in MrpC binding sites which likely lead to disruption of MrpC NAR. In general, NAR motifs are thought to contribute to an organism’s robustness against mutational perturbations (Marciano et al., 2014). For example, mutations in NAR TF genes are tolerated at a higher rate than in non-NAR TF genes, likely because alterations in NAR TF activity are automatically compensated by changes in the TF self-expression (Marciano et al., 2016). Mutations in a self-promoter, however, can alter the steady state levels of the TF without a corresponding compensation in binding affinity to other target promoters (Kozuch et al., 2020). In a developmental transcriptional network with multiple interconnected regulatory motifs (Supplementary Figure S6C), a perturbed NAR motif leading to increased cell-to-cell variation could lead to subpopulations of cells which trigger or bypass connected regulatory motifs to induce novel cell fate trajectories. Given that *M. xanthus* development is heavily influenced by a number of factors independent of genetic regulatory networks, including physical factors (e.g., surface characteristics) and mechanical properties (e.g., coarsening processes) (Bahar et al., 2014; Liu et al., 2019; Guzman-Herrera et al., 2020; Ramos et al., 2021), it is likely that emerging phenotypes could be drastically different depending on specific environmental conditions or difference in initial population density, leading to eventual selection of beneficial phenotypes perhaps optimized for specific environmental niches.

## Data availability statement

The raw data supporting the conclusions of this article will be made available by the authors, without undue reservation.

## Author contributions

MM: Conceptualization, Writing – original draft, Writing – review & editing, Investigation. PH: Conceptualization, Writing – original draft, Writing – review & editing, Formal analysis, Funding acquisition, Supervision.
